# Design and Modelling of MEMS Vibrating Internal Ring Gyroscopes for Harsh Environments

**DOI:** 10.3390/s24175854

**Published:** 2024-09-09

**Authors:** Waqas Amin Gill, Ian Howard, Ilyas Mazhar, Kristoffer McKee

**Affiliations:** Department of Mechanical Engineering, Curtin University, Perth, WA 6845, Australia

**Keywords:** Microelectromechanical Systems vibrating ring gyroscope, inertial sensors, harsh environments, mode matching, space applications, inertial measurement unit (IMU), ring resonator

## Abstract

This paper presents a design, model, and comparative analysis of two internal MEMS vibrating ring gyroscopes for harsh environmental conditions. The proposed design investigates the symmetric structure of the vibrating ring gyroscopes that operate at the identical shape of wine glass mode resonance frequencies for both driving and sensing purposes. This approach improves the gyroscope’s sensitivity and precision in rotational motion. The analysis starts with an investigation of the dynamic behaviour of the vibrating ring gyroscope with the detailed derivation of motion equations. The design geometry, meshing technology, and simulation results were comprehensively evaluated on two internal vibrating ring gyroscopes. The two designs are distinguished by their support spring configurations and internal ring structures. Design I consists of eight semicircular support springs and Design II consists of sixteen semicircular support springs. These designs were modelled and analyzed using finite element analysis (FEA) in Ansys 2023 R1 software. This paper further evaluates static and dynamic performance, emphasizing mode matching and temperature stability. The results reveal that Design II, with additional support springs, offers better mode matching, higher resonance frequencies, and better thermal stability compared to Design I. Additionally, electrostatic, modal, and harmonic analyses highlight the gyroscope’s behaviour under varying DC voltages and environmental conditions. Furthermore, this study investigates the impact of temperature fluctuations on performance, demonstrating the robustness of the designs within a temperature range from −100 °C to 100 °C. These research findings suggest that the internal vibrating ring gyroscopes are highly suitable for harsh conditions such as high temperature and space applications.

## 1. Introduction

In the modern era of technological advancement, where miniaturization, energy efficiency, and high-performance devices are integral in many applications, Microelectromechanical Systems (MEMS) have become an important part of many advanced smart electronic systems [1,2,3,4]. These microscale devices are made up of tiny mechanical and electrical structures on a single electronic chip, enabling their use in a wide spectrum of applications ranging from healthcare to automotive to space applications.

MEMS devices are attractive due to their easy integration into a broad range of fields, including the biomedical, space, automotive, and consumer electronics industries. MEMS inertial sensors are crucial sensors in inertial measurement units (IMUs), which have been extensively used in inertial navigation [5] for space applications [6,7]. The vibrating gyroscope is an integral component of the inertial measurement unit (IMU), which is used to detect and control rotational motion in various technologies. There are different types of MEMS vibrating gyroscopes that exist. However, MEMS vibrating ring gyroscopes possess many advantages over other vibrating gyroscopes because of their identical mode of vibration and symmetrical design structure.

MEMS vibrating ring gyroscopes are critical inertial sensors for a wide range of applications that demand high precision, robustness, and compact sizes [8,9,10]. In aerospace and aviation [11,12], these gyroscopes are integral to inertial navigation systems, providing essential data for flight control and navigation under extreme conditions such as high altitudes, rapid thrusts, and temperature fluctuations. Similarly, they are extensively utilized in guided missiles, UAVs, and other military systems. Their ability to maintain accuracy in harsh environments, including extreme temperatures and high vibrations, is vital for mission success in the defence sector [13,14].

MEMS gyroscopes play a vital role as sensors in the automotive industry [15,16]. They are instrumental in enhancing the safety and efficiency of advanced driver-assistance systems (ADAS) and autonomous vehicles. They ensure stable operation even in challenging driving conditions. Furthermore, they play a crucial role in the monitoring of industrial processes and the environment [17]. They help to optimize the performance of heavy machinery and infrastructure, as well as in seismic sensors and weather monitoring systems, where reliability under harsh environmental conditions is vital.

Moreover, MEMS vibrating ring gyroscopes are widely used in consumer electronics, such as smartphones and gaming devices [18]. These inertial sensors enable features like motion detection and image stabilization. In space exploration, these gyroscopes must withstand the challenging conditions of vacuum, radiation, and extreme temperature changes. They are essential for maintaining the orientation and stabilization of the instruments in satellites and space probes [19].

The main objective of this research is to design and showcase an innovative design for a MEMS vibrating ring gyroscope for harsh conditions. As previously mentioned, the MEMS vibrating ring gyroscope operates at two identical resonance frequencies for driving and sensing mechanisms [20,21,22,23,24]. The ring structure in the MEMS vibrating ring gyroscope allows for a unique implementation of identical vibrational modes for both the driving and sensing mode systems [25,26,27,28]. This symmetric design offers enhanced gyroscope sensitivity and precision in detecting rotational rates and motions [9,29,30].

If the vibrating ring gyroscope operates at the same resonance frequencies for driving and sensing, it exhibits optimal performance. However, there are many practical anomalies encountered by MEMS designers during the design and fabrication phases of these MEMS gyroscopes. The encountered errors in the processes affect the performance characteristics of the MEMS gyroscopes [31,32,33]. These gyroscopes are highly susceptible to changes in environmental conditions and micro-fabrication processing errors [32,34,35,36,37]. These errors result in a mode mismatch in the resonance frequencies, leading to a discrepancy between the frequencies of the driving and sensing modes. The MEMS vibrating ring gyroscope is a popular design of vibrating gyroscopes. It consists of a ring resonator supported by semicircular springs, which are attached to the anchors to support the whole structure of the vibrating gyroscope. The symmetric design structure of the MEMS vibrating ring gyroscope provides high thermal stability and a low mode mismatch resonance frequency value, which are crucial for its operation in harsh environments. However, traditional vibrating ring gyroscopes’ sensitivity is compromised when exposed to high-temperature fluctuations and unwanted atmospheric vibrations.

To compensate for the above shortcomings of traditional vibrating ring gyroscopes, we investigated two designs of MEMS vibrating internal ring gyroscopes that incorporate an internal ring with semicircular support springs attached to the externally placed anchor. The analysis starts with the motion equations of the MEMS vibrating ring gyroscope. Furthermore, a thorough analysis of the design geometry, meshing methodology, and the modelled results and comparisons was performed on Designs I and II of the selected MEMS vibrating internal ring gyroscope. The design of the internal ring gyroscope offers a higher desired resonance frequency with a lower mode mismatch value than traditional external ring gyroscopes.

The later sections of this paper discuss a thorough investigation of the temperature analyses and mode matching of the vibrating internal ring gyroscopes. The temperature analyses include thermal stresses, thermal strains, and thermal deformations of the vibrating structure when exposed to a temperature range from −100 °C to 100 °C. Furthermore, the two designs were analyzed for mode matching and were compared with the traditional external ring gyroscope. An FEA-based model is implemented in Ansys 2023 R1 and compares the results of the two selected MEMS vibrating internal ring gyroscopes.

## 2. Design Analysis

A MEMS vibrating ring gyroscope works on the principle of vibrating structure motion and the Coriolis force with a symmetric design structure. The design comprises the ring-shaped drive mode oscillator that generates and maintains a constant elliptical vibration mode along the driving axis. The drive mode system consists of the ring resonator structure, beam, and support anchor structures with driving electrodes. There is a sense mode system about 45 degrees from the drive mode system. The sense mode system consists of the ring resonator structures, beams, and support anchor structures with sensing electrodes. The sensing electrodes are designed to detect the developed Coriolis forces because of the rotation applied to the system.

**Figure 1:**
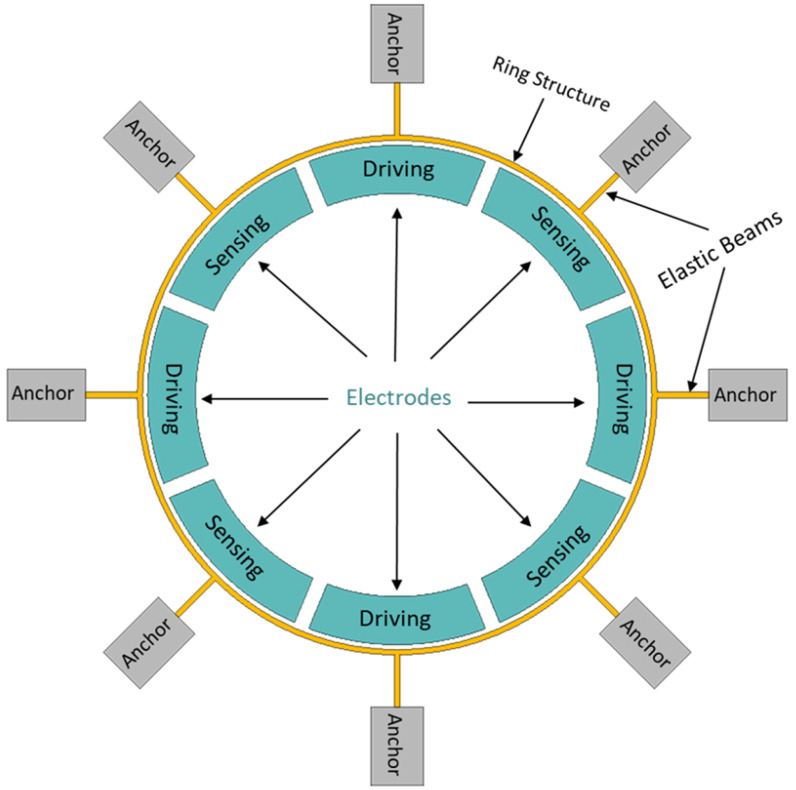
A schematic illustration of a dynamic system of a MEMS vibrating ring gyroscope.

The dynamic operations of a MEMS vibrating ring gyroscope involve the monitoring analysis of the Coriolis forces detected by the sensing electrodes. These Coriolis forces result from the vibrating ring interacting with externally applied angular rates, allowing for the precise measurement of rotation and orientation changes. The design and implementation of these vibrating ring gyroscopes make them ideal candidates for applications requiring high accuracy and reliability in harsh conditions. A typical MEMS vibrating ring gyroscope dynamic system is schematically illustrated in Figure 1.

**Figure 2:**
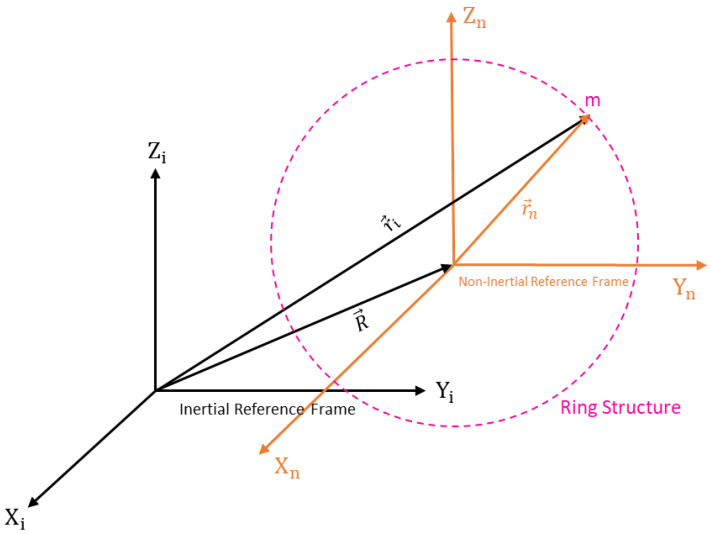
An illustration of the reference frames in terms of a ring resonator.

A ring structure, eight straight beams, and eight support anchors are placed around the set of driving and sensing electrodes, as shown in Figure 1. In a MEMS vibrating ring gyroscope, the dynamics and fundamentals of operation are centred on the Coriolis force. The vibrating ring gyroscope operates in a rotating reference frame. Its dynamics can be determined through the rotation-induced Coriolis force acting on the ring observed in the inertial frame. The vibrating ring gyroscope dynamics regarding the inertial reference frame are shown in Figure 2. The complete derivation of the reference frame related to the vibrating ring resonator is presented in our recent paper [38].

The position vector $\vec{x_{i}}$ related to the inertial reference is shown in the equation below.

(1) $${\vec{x}}_{i}=\vec{R}+{\vec{x}}_{n}$$

By further solve and differentiate the above equation when the rotation comes into place, we can determine the acceleration of the inertial reference frame.

(2) $$\ddot{x_{i}}=\ddot{\vec{R}}+\ddot{{\vec{x}}_{n}}+\dot{\vec{\Omega }}\times \vec{x_{n}}+2\vec{\Omega }\times \dot{{\vec{x}}_{n}}+\vec{\Omega }\times \left(\vec{\Omega }\times {\vec{x}}_{n}\right)$$

We will put Equation (2) into Newton’s second law of motion to find out the forces in the MEMS vibrating ring gyroscope.

(3) $$F=m\left[\ddot{\vec{R}}+\ddot{{\vec{x}}_{n}}+\dot{\vec{\Omega }}\times \vec{x_{n}}+2\vec{\Omega }\times \dot{{\vec{x}}_{n}}+\vec{\Omega }\times \left(\vec{\Omega }\times {\vec{x}}_{n}\right)\right]$$

where *F* is the force which is applied to the system, $\ddot{x_{i}}$ is the acceleration experienced by the system, $\ddot{\vec{R}}+\ddot{{\vec{x}}_{n}}+\dot{\vec{\Omega }}\times \vec{x_{n}}$ is the acceleration that is experienced in the non-inertial reference frame, $\vec{\Omega }$ is the angular velocity, and $2\vec{\Omega }\times \dot{{\vec{x}}_{n}}$ is the Coriolis force of the gyroscope system.

### 2.1. Motion Equations of Internal Vibrating Ring Gyroscope

**Figure 3:**
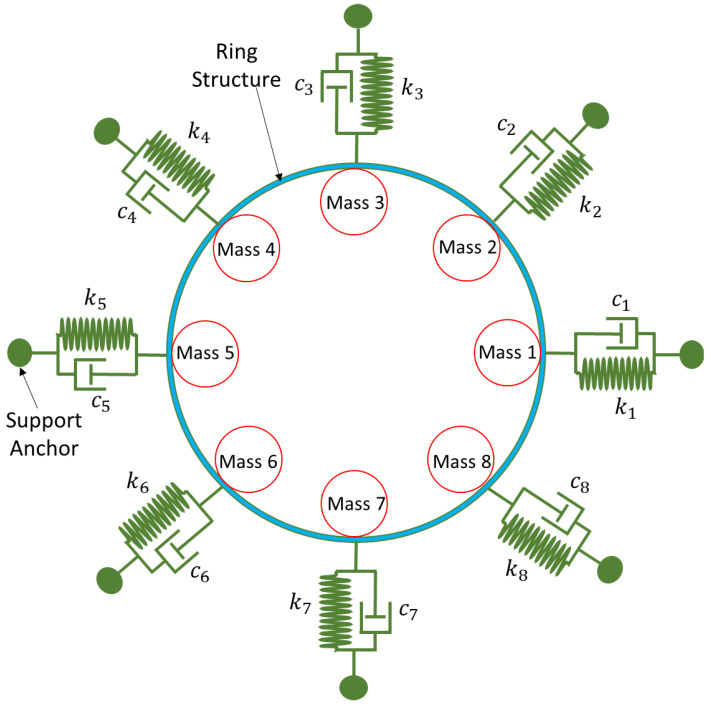
A resonant structure model for the MEMS internal ring gyroscope shows with separate eight masses.

Let us consider the dynamics of the complete MEMS internal vibrating ring gyroscope system, including externally placed semicircular support beams. The resonant structure of an internal ring gyroscope is schematically shown in Figure 3. The illustration shows eight spring mass damping systems that contribute to the main structure of the vibrating ring gyroscope system.

**Figure 4:**
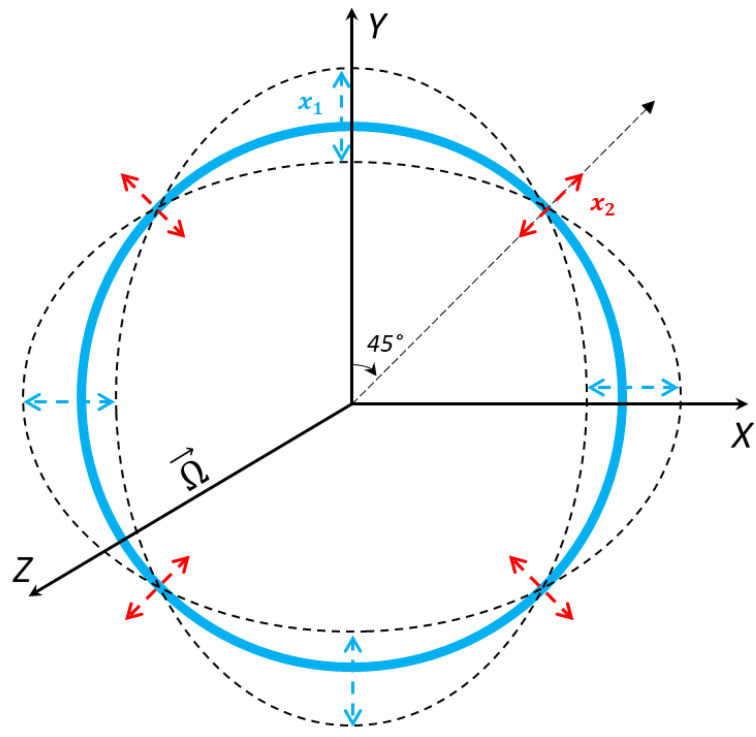
An elliptical mode of vibration for a ring resonator and displacement illustration for primary and secondary vibrational modes.

The internal ring structure connects eight semicircular support springs, and the vibrating structure is attached to the externally placed support anchors. The ring structure oscillates in identical pairs of mode vibrations. We will consider the identical elliptical mode of vibrations. Its dynamic representation is shown in Figure 4, with displacements occurring during oscillation.

**Figure 5:**
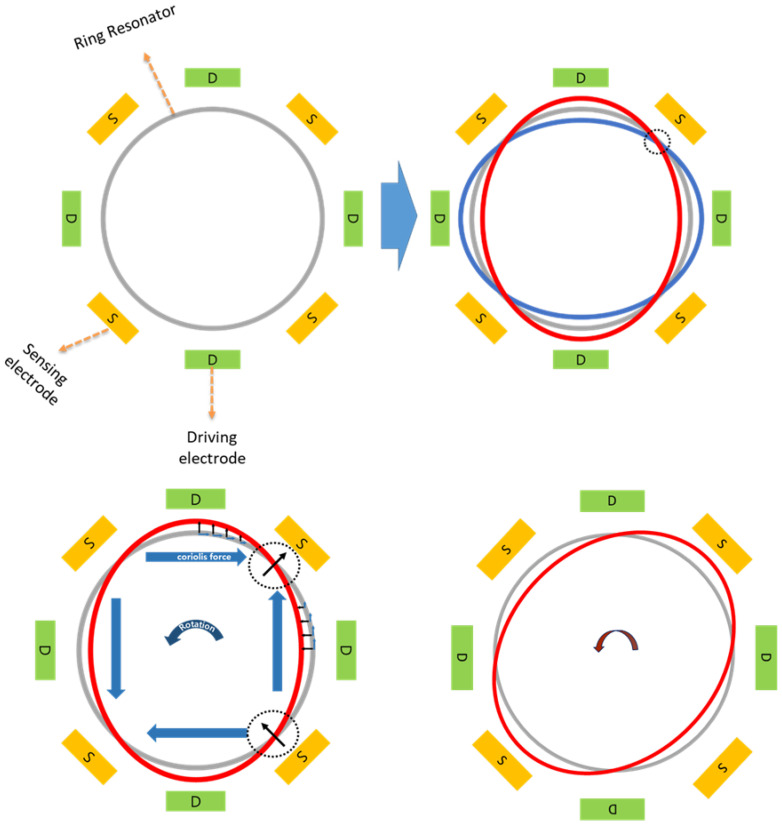
Driving and sensing mechanism of a ring resonator.

The inplane displacement of the vibrating ring resonator is shown in Figure 4. Each small segment of the MEMS vibrating ring gyroscope system considers two degrees of freedom. When the excitation is provided on the driving axis, the ring resonator oscillates as an elliptical mode of vibrations along two orthogonal axes. When the rotation is applied on the *z*-axis, the Coriolis force in the reaction is developed by the primary mode of vibration, and that Coriolis force transfers the identical elliptical mode at 45 degrees between the initially two orthogonally drive axes. The ring’s dynamic behaviour is depicted in Figure 5.

When a ring oscillates in elliptical mode 2θ, it experiences radial $x_{1}$ and tangential $x_{2}$ displacements, as shown in Figure 4. The respective displacements are written as Equations (4) and (5).

(4) $$x_{1}=A_{1}\cos2\theta +A_{2}\sin2\theta$$

(5) $$x_{2}=\frac{A_{1}}{2}\sin2\theta -\frac{A_{2}}{2}\sin2\theta$$

To find the equations of motion for the MEMS vibrating ring gyroscope, we will consider the Lagrange equation of motion.

(6) $$\frac{d}{dt}\left(\frac{\partial K_{E}}{\partial {\dot{e}}_{i}}\right)-\frac{\partial K_{E}}{\partial e_{i}}-\frac{\partial R_{E}}{\partial {\dot{e}}_{i}}-\frac{\partial (U_{R}+U_{SS})}{\partial e_{i}}=\frac{{\partial E}_{i}}{\partial e_{i}},\;(i=1,2,\ldots ,n)$$

Here, $K_{E}$ is the kinetic energy, $R_{E}$ is the damping energy, $U_{R}$ is the strain energy, $U_{SS}$ is the strain energy of the support springs, and E is the applied force to the gyroscope system.

#### 2.1.1. Kinetic Energy of the System

Let us start to evaluate the equations of motion. First, we determine the system’s kinetic energy. In addition to finding the absolute velocities $v_{1},\;v_{2}$ from the absolute displacements of the gyroscope from the central axes, the ring displacements $x_{1},\;x_{2}$ are described in Equations (4) and (5). The kinetic energy of the ring resonator is written as follows:

(7) $$K_{E}=\frac{1}{2}A\rho R\int_{0}^{2\pi }({v_{1}}^{2}+{v_{2}}^{2})d\theta$$

where R is the radius, A is the cross-sectional area of the ring structure, and ρ is the density of the material. By solving Equation (7), we evaluate the simplified kinetic energy equation of the system as presented in Equation (8). Here, $A_{1}$ and $A_{2}$ are the general coordinates of the ring structure.

(8) $$K_{E}=\frac{1}{2}m\left[\frac{5}{8}{\left(\frac{dA_{1}}{dt}\right)}^{2}+\frac{5}{8}{\left(\frac{dA_{2}}{dt}\right)}^{2}\right]$$

Equation (8) describes the kinetic energy equation of the ring resonator related to the vibrating ring gyroscope. The respective equation accounted for the ring’s rigid motion and elliptical vibration modes.

#### 2.1.2. Strain Energy of the Ring Structure System

The strain energy of the system is considered to be the ring structure’s elastic strain energy as represented by Hook’s law. The elastic strain energy of the ring resonator is given as Equation (9).

(9) $$U_{R}=\frac{ER}{2}\int_{0}^{2\pi }\int \epsilon^{2}dAd\theta$$

**Figure 6:**
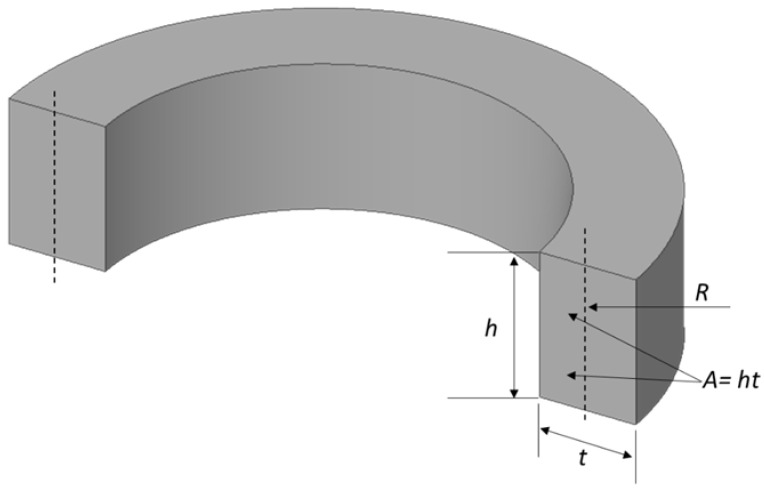
A cross-sectional view of the ring structure with centreline radius R and other design parameters.

The cross-sectional view of the ring structure is shown in Figure 6, where R is the radius of the ring from the centreline, h is the height of the ring, t is the thickness of the ring, ϵ is the normal strain of the ring, and A is the cross-sectional area of the ring. The normal strain of the ring structure is represented as Equation (10).

(10) $$\epsilon =\frac{1}{R}\left[-x_{1}+\frac{\partial x_{2}}{\partial \theta }-\frac{x}{R}\frac{\partial }{\partial \theta }\left(x_{2}+\frac{\partial x_{1}}{\partial \theta }\right)\right]$$

Let us consider, the inextensionality of the ring makes ϵ=0 at x=0. Equation (10) can be written as $x_{1}=\frac{\partial x_{2}}{\partial \theta }$.

After solving the above equations, the ring’s strain energy, which depicts elliptical oscillation modes, can be represented as Equation (11).

(11) $$U_{R}=\frac{EI\pi }{2R^{3}}\left(9{A_{1}}^{2}+9{A_{2}}^{2}\right)$$

#### 2.1.3. Strain Energy of Support Springs

The strain energy for the support springs in the gyroscopic system is quite important, as it completes the spring mass damping system. This section discusses and derives the strain energy equation of support springs for a ring gyroscope. The size of the support springs is relatively small compared to the size of the ring, so we assume the kinetic energy of the support spring is negligible or zero.

**Figure 7:**
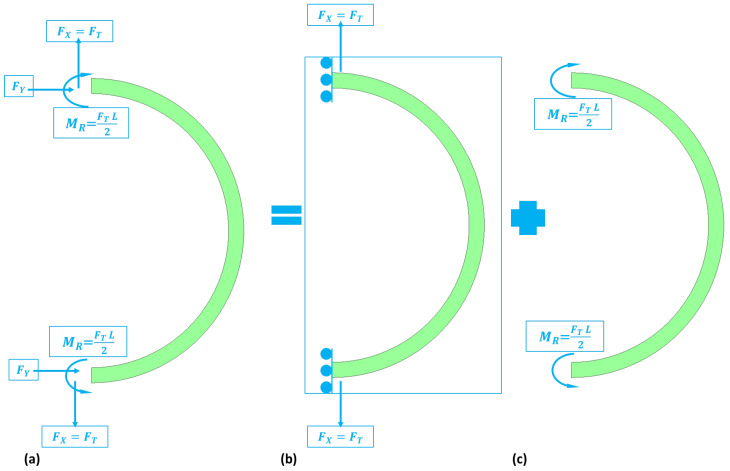
An illustration of the force’s reactions on the semicircular support springs (**a**) forces and moments experienced by semicircular support springs (**b**) normal forces (**c**) moments.

To study the dynamics of the support springs, we consider the forces acting on the springs when it is in operation. The semicircular support spring experiences tangential and radial displacements due to normal and shear forces. Therefore, the tangential and radial stiffnesses for the semicircular support spring are required to determine the motion equation of the MEMS vibrating ring gyroscope. The forces experienced by the support spring are schematically presented in Figure 7.

To find out the tangential and radial stiffnesses of the support spring, the strain energy equation considered is written as Equation (12).

(12) $$U_{s}=\int \frac{1}{2}\sigma \varepsilon dV$$

Here, $U_{s}$ is strain energy developed into the support spring due to the normal force and bending moment, σ is the stress, and ε is the strain developed due to the bending moment. The equation is further solved and written as Equation (13).

(13) $$U_{s}=\int_{0}^{L}\frac{1}{2}\frac{M^{2}}{EI_{c}}\;dx$$

Here, E is the Young’s modulus, *M* is the bending moment experienced by the support spring, *L* is the length of the support spring, and $I_{c}$ is the moment of inertia.

The complete strain energy equation is derived from our previously published article [39]. The final strain energy equation for the semicircular support springs is given below. Here, $F_{T}$ is the applied normal force.

(14) USS=∫0π21EIcM−FTr2(1−cosθ)2rdθ

The stiffness constant for the semicircular support spring is given as Equation (15), where r is the beam radius, w is the width of the beam, and h is the height of the beam.

(15) 1kSS=1kT+1kR=2r3EIc∫0π2cosθ2−1π2dθ+πrL24EIc

Here, $k_{SS}$ is the stiffness constant for the semicircular support spring, and $k_{T}$ and $k_{R}$ are the tangential and radial stiffnesses of the semicircular support spring.

If the number of support springs increases, they will enhance the resonance frequency and stability of the ring’s oscillations. Equation (16) represents the total strain energy developed by the support springs in the vibrating ring gyroscope, where n is the number of support springs and i represents the position of the support beams attached to the ring.

(16) $$U_{SS}=\sum_{i=1}^{n}\frac{{x_{1}}^{2}}{2}k_{R}+\sum_{i=1}^{n}\frac{{x_{2}}^{2}}{2}k_{T}$$

To obtain the more simplified strain energy Equation (17), Equations (4) and (5) are substituted into Equation (16) for the total number of support springs in the gyroscope structure.

(17) $$U_{SS}=\frac{k_{R}}{2}\left(4{A_{1}}^{2}+4{A_{2}}^{2}\right)+\frac{k_{T}}{2}\left({A_{1}}^{2}+{A_{2}}^{2}\right)$$

#### 2.1.4. Damping Energy of the System

To design a MEMS vibrating ring gyroscope, a consideration of thermoelastic damping is quite important. This method involves thermal expansion and mechanical strain, which causes the energy to dissipate in the gyroscope. The selection of materials for the gyroscope is quite crucial, as different materials exhibit different damping characteristics. The Rayleigh damping function also plays an important role in gyroscope performance. A comprehensive analysis of the Rayleigh damping function reveals how the impact of geometrical dimensions and material properties affect the damping characteristics, alongside structural and viscous damping characteristics, provide a broader perspective in sensor applications.

The damping energy for MEMS vibrating ring gyroscope in the vacuum is usually thermoelastic damping. The Rayleigh damping function for the MEMS vibrating ring gyroscope is presented in Equation (18). Note that the Rayleigh function equation here is only for identical modes of vibrations.

(18) $$R_{E}=\frac{1}{2}\left[c\left({\left(\frac{dA_{1}}{dt}\right)}^{2}+{\left(\frac{dA_{2}}{dt}\right)}^{2}\right)\right]$$

#### 2.1.5. Electrostatic Energy of the System

The capacitive electrodes are designed to be placed around the ring structure. These electrodes are used for electrostatic actuation and capacitive detection in the gyroscope system. The electrodes make parallel plate capacitors with the ring resonator. These capacitive electrodes exert electrostatic forces on the ring resonator to oscillate the ring at the set resonance frequency. Therefore, the ring structure acts as a parallel plate capacitor with the surrounded electrode. The voltages across the capacitors are labelled as outer voltage and inner voltage. The electrostatic energy of these parallel plate capacitors can be expressed as Equation (19).

(19) $$dE=\frac{\varepsilon_{0}\varepsilon V^{2}}{2d}dA$$

where E is the electrostatic energy of the system, $\varepsilon_{0}$ is the permittivity of free space, ε is the permittivity of the gyroscope material, d is the spacing between the two parallel plate electrodes, and $dA_{c}$ is the differential capacitor area. The gap between the two parallel plate capacitors varies and depends on the nominal gap denoted as $d_{g}$ and the radial displacement $x_{1}$.

(20) $$d=d_{g}\pm x_{1}(\theta )$$

Let us substitute Equation (20) into Equation (19).

(21) $$dE=\frac{\varepsilon_{0}\varepsilon V^{2}}{2d_{g}}\left(1+\frac{x_{1}}{d_{g}}+{\left(\frac{x_{1}}{d_{g}}\right)}^{2}+{\left(\frac{x_{1}}{d_{g}}\right)}^{3}+\ldots \right)dA_{c}$$

(22) $$dE=\frac{\varepsilon_{0}\varepsilon V^{2}}{2d_{g}}\sum_{k=0}^{N}{\left(\pm 1\right)}^{k}{\left(\frac{x_{1}}{d_{g}}\right)}^{i}dA_{c}$$

A differential capacitor area is composed of $lr_{i}d\theta$ for the inner capacitor or $lr_{o}d\theta$ for the outer capacitor, each having a different mean radius denoted as inner or outer. The electrodes are an integral part of the gyroscope and are placed nearby to create the small circumferential gaps. The electrostatic energy stored in each part of the inner and outer capacitor is determined by integrating Equation (22). The total electrostatic energy can be determined by the equation given below, where $A_{e}=\frac{\varepsilon_{0}\varepsilon \;l}{2d_{g}}\;$is the area of the electrode.

(23) $$E=A_{e}\sum_{k=0}^{N}\left(\frac{r_{o}{V_{o}}^{2}+{\left(-1\right)}^{k}r_{i}{V_{i}}^{2}}{{d_{g}}^{k}}\int_{0}^{2\pi }{x_{1}}^{k}d\theta \right)$$

The nonlinear electrostatic force $F_{{nl}_{i}}\;$can be determined by using Equation (24).

(24) $$F_{{nl}_{i}}=-∆A_{e}\sum_{k=3}^{N}\left(\frac{k\left(r_{o}{V_{o}}^{2}+{\left(-1\right)}^{k}r_{i}{V_{i}}^{2}\right)}{{d_{g}}^{k}}\right)\left(\int_{0}^{2\pi }{x_{1}}^{k-1}\cos\left(i\theta \right)d\theta +\int_{0}^{2\pi }{x_{1}}^{k-1}\sin\left(i\theta \right)d\theta \right)$$

The linear electrostatic stiffness is different than the nonlinear electrostatic stiffness. The linear electrostatic stiffness can be determined as Equation (25).

(25) $$k_{e}=-2\pi A_{e}\frac{r_{o}{V_{o}}^{2}+r_{i}{V_{i}}^{2}}{{d_{g}}^{2}}$$

The total stiffness of the system is affected by the linear electrostatic forces applied by the outer and inner capacitors, resulting in a softening effect. The softening effect accelerates as the electrode voltages rise and the gyroscope system’s total stiffness value becomes negative. This results in the instability of the gyroscope system if the voltage reaches a certain threshold. Several observations relevant to the nonlinear electrostatic force are described below.

In order to eliminate gyroscope coupling mechanisms, it is important to apply the electrode voltages so that $r_{o}{V_{o}}^{2}=r_{i}{V_{i}}^{2}$. This will cause any even power terms in the generalized coordinates in Equation (24) to cancel out.

The nonlinear electrostatic force’s expansion shows a softening or hardening effect in the gyroscope system. For example, hardening effects happen when $r_{i}{V_{i}}^{2}>r_{o}{V_{o}}^{2}$.

Let us consider Lagrange’s equation to determine the motion equations for the vibrating ring gyroscope.

(26) $$\frac{d}{dt}\left(\frac{\partial K_{E}}{\partial {\dot{e}}_{i}}\right)-\frac{\partial K_{E}}{\partial e_{i}}-\frac{\partial R_{E}}{\partial {\dot{e}}_{i}}-\frac{\partial (U_{R}+U_{SS})}{\partial e_{i}}=\frac{{\partial E}_{i}}{\partial e_{i}}$$

Previously derived energy equations will be substituted into Lagrange’s equation, further simplifying the equations of motion. As a result, we have two equations of motion for the elliptical shape of resonant modes.

(27) $$m_{G}\ddot{A_{1}}+c_{G}\dot{A_{1}}+k_{G}A_{1}=F_{A-}F_{{nl}_{1}}$$

(28) $$m_{G}\ddot{A_{2}}+c_{G}\dot{A_{2}}+k_{G}A_{2}=F_{A-}F_{{nl}_{2}}$$

These equations are developed for the elliptical mode of vibrations for the MEMS vibrating ring gyroscope. Here${\;m}_{G}$, $c_{G}$, and $k_{G}$ are the mass, damping coefficient, and stiffness constant for the elliptical vibration mode. The coordinates $A_{1,\;}\;A_{2\;}$ represent 2θ modes of vibrations for a ring gyroscope. Here, $F_{A}$ is the applied base excitation force to the externally placed support anchors and $F_{nl}$ is the nonlinear force that depends on the modal mass of the particular vibration mode.

### 2.2. Modelling of the Proposed Internal Vibrating Ring Gyroscopes

In the finite element analysis (FEA) of the MEMS vibrating ring gyroscopes, several key modelling steps were carefully implemented to ensure accurate results. The analysis was conducted using Ansys 2023 R1. It started with the precise design geometry of the vibrating ring gyroscope structures, which includes the ring and semicircular support springs. Fine meshes consisting of tetrahedral and hexahedral elements were applied, particularly in critical areas such as the semi-circular support springs and resonator ring structure. A mesh convergence study ensured that further mesh refinement did not significantly alter the results, thereby minimizing discretization errors. Material properties for silicon, such as Young’s modulus (170 GPa), Poisson’s ratio (0.28), and density (2330 kg/m^3^), were incorporated, along with temperature-dependent properties to simulate the vibrating ring gyroscope’s performance under extreme thermal conditions.

The boundary conditions were applied with fixed supports at the external anchors to hold the structural layer to the substrate, which allows for the ring and springs to vibrate freely. The simulation methodology involved a modal analysis to determine the natural frequencies and mode shapes, followed by a harmonic response analysis to evaluate the vibrating ring gyroscope’s behaviour under external vibrations, particularly at resonance frequencies. In this comprehensive FEA study, two designs of MEMS vibrating internal ring gyroscopes are modelled and proposed. Ansys 2023 R1 Workbench software was used for the FEA study. These gyroscopes are designed as a single layout for simple fabrication process of silicon on the insulator (SOI) process. The SOI process is explained in our published conference proceedings [40]. The material properties, geometrical dimensions, and boundary conditions followed as per the SOI process.

This FEA study aims to evaluate the performance of the two proposed gyroscopes, including static and dynamic modelling of the vibrating ring gyroscopes. The initial analysis targets the electrostatic actuation effect on the gyroscope ring structure, then finds the modal resonance frequencies, followed by a harmonic analysis of the ring gyroscope. The initial modal results provide a detailed analysis of the vibrational behaviour of the vibrating ring gyroscopes, emphasizing the identical elliptical mode of vibrations. The modal analysis is important for guiding potential design improvements and further gyroscope efficiency and reliability. A critical modelling study on the harmonic analysis is necessary to understand the response amplitude of the vibrating ring gyroscope under two identical modes of resonance frequencies.

Furthermore, simulation modelling of the vibrating ring gyroscope behaviour in harsh environmental conditions was used to assess their stability and reliability. Particular attention is given to the mode matching of two resonance frequencies and temperature variations on the performance of the gyroscope. The simulation analysis is essential to assess the performance of the gyroscope, ensuring that drive and sense mode frequencies are optimally matched for higher sensitivity and accuracy. Temperature variation is another important factor for MEMS devices. MEMS gyroscopes can be sensitive to temperature variations, affecting their performance. Due to their symmetric design geometry, the MEMS vibrating ring gyroscope provides excellent results on temperature variation applications. The simulation includes various temperature ranges to evaluate the structure’s thermal stability and other related mechanical properties.

#### 2.2.1. Design Geometry

In modern-day smart devices, the precision of the device is an integral factor, particularly in inertial sensing. Therefore, the design geometry of a MEMS vibrating ring gyroscope regarding preciseness and accuracy is quite important. Two designs were selected and proposed for the MEMS vibrating internal ring gyroscope. Semicircular support springs with externally placed anchors provide structural support to the resonator ring structure. The resonator ring’s radius is designed to achieve a targeted resonance frequency of 50 kHz.

**Figure 8:**
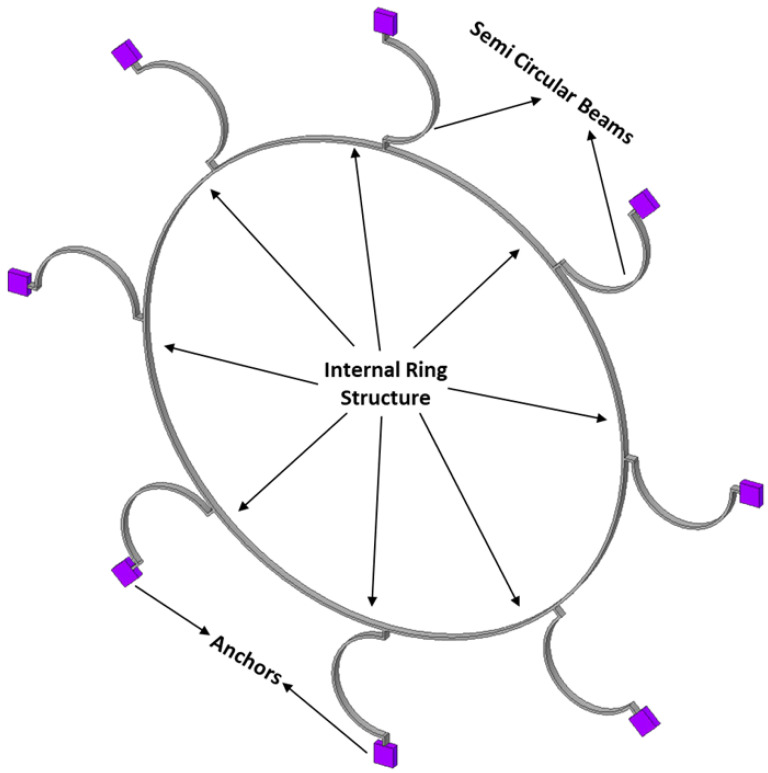
Design I: MEMS vibrating internal ring gyroscope with eight semicircular support springs.

**Design I:** The Design I of the MEMS vibrating ring gyroscope is shown in Figure 8. The design can be identified by its eight semicircular support springs. Each measures 210 µm in radius and 10 µm in thickness. The gyroscope design has a ring radius of 1000 µm while maintaining a structure height of 100 µm and a ring thickness of 10 µm. The externally placed anchor supports are designed with dimensions of 80 µm by 80 µm to ensure stability and support throughout the whole vibrating gyroscope structure.

The support springs are designed in a semicircular shape to maximize mechanical stability and efficiency in energy usage. The design emphasizes the balance between flexibility and stability by reducing the number of springs. The symmetrical design approach of the springs surrounding the central ring provides an even distribution of stress that is particularly advantageous in unwanted vibration applications. The symmetry of the structure ensures that the operational frequencies remain consistent even when exposed to various temperature ranges. The design parameters are listed in Table 1.

**Figure 9:**
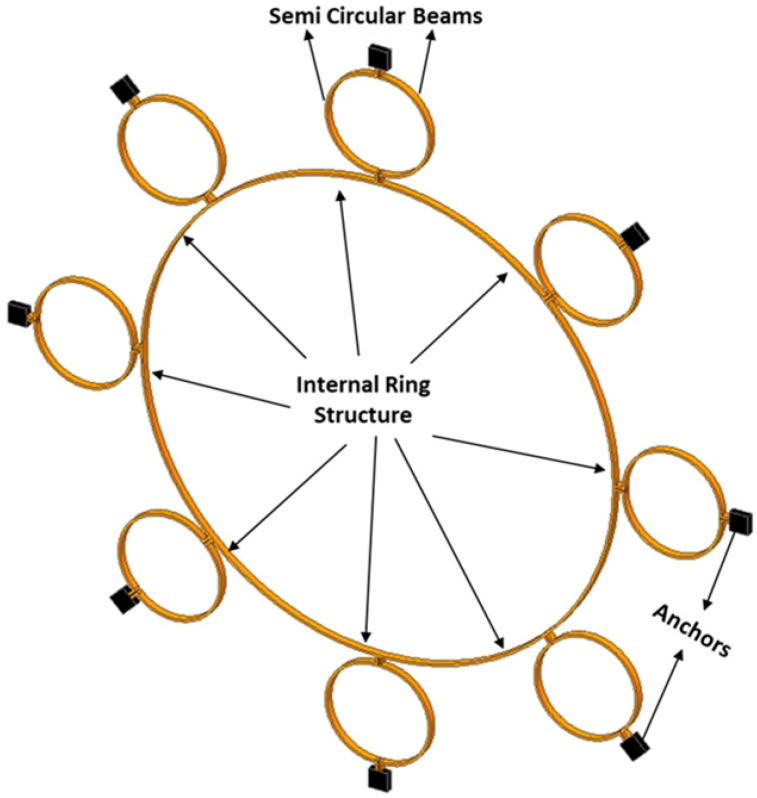
Design II: MEMS vibrating internal ring gyroscope with sixteen semicircular support springs.

**Design II:** Design II improves the gyroscope’s structure by incorporating sixteen semicircular support springs. Adding more support springs enhances the design’s capacity to withstand environmental changes, such as external vibration fluctuations due to various temperature ranges [7]. The design features are the same as Design I; the only difference is the addition of more semicircular support springs. It also supports a central ring with a radius of 1000 µm, a height of 100 µm, and a thickness of 10 µm. Design II is shown in Figure 9.

Design II incorporates double the number of support springs compared to Design I. This results in a more uniform distribution of stresses. This is particularly important in conditions with significant fluctuations due to external vibrations and temperature changes. The symmetrical design configuration of the gyroscope ensures that temperature changes are equally distributed, helps to maintain gyroscope accuracy, and prevents any mode mismatch in the resonant frequencies caused by thermal expansion or contraction. The design parameters for Design II are listed in Table 2.

#### 2.2.2. Mesh Generation

The objective of the meshing is to create a mesh model that accurately represents the physical model of the complex design of a MEMS vibrating ring gyroscope. To achieve the desired resonance frequency and mode matching for harsh environments, it is a must that the mesh captures the high resolution of the gyroscope geometry, especially around the ring and semicircular support spring structures. The meshing approach was selected based on the vibrating ring gyroscope’s geometric complexities. In both Design I and Design II, the meshing procedure started by preparing the geometry model to eliminate minor imperfections that may affect the creation of the mesh. An important emphasis was placed on the ring and semicircular support spring structures that are crucial for ensuring the stability and performance of the gyroscope.

The mesh generation used a mixed approach of incorporating both tetrahedral and hexahedral elements to maximize the advantages offered by each element type [41]. Tetrahedral elements were used to mesh the ring resonator and semicircular support springs. They possess a complex curvature and require flexibility in the mesh structure. The tetrahedral mesh adapts to complex geometries and upholds quality throughout the model. The externally placed anchor structures have a reasonably simple structure. The respective structure was divided into hexahedral elements to decrease the number of elements and improve the simulation performance.

Tetrahedral meshing was used for the ring and semicircular support springs because of the complex geometric features of the ring gyroscope. A patch-conforming tetrahedron mesher was used for high-quality geometries. The layered tetrahedrons allowed for adding multiple layers of elements to capture the effects of the boundary layer.

Hexahedral meshing was used for the externally placed anchors, where fewer elements were needed to run the simulation programme effectively and efficiently. The swept meshes in the layered hexagonal mesh are used for the minimum mechanical energy usage, and the multizone feature is utilized for multiple regions in the anchor support.

#### 2.2.3. Electrostatic Modelling

**Figure 10:**
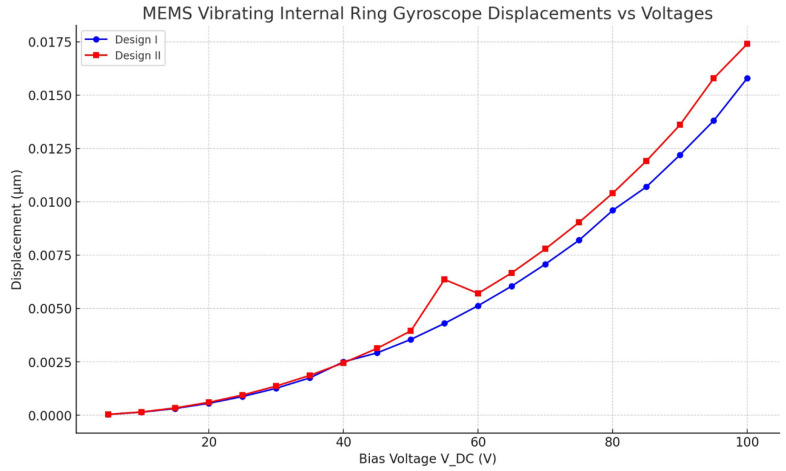
Electrostatic actuation modelling results for MEMS vibrating internal ring gyroscope for Design I and Design II.

Electrostatic modelling was investigated to determine how a structure responds to a biassed voltage static load. It is important to estimate the stiffness of a MEMS vibrating ring gyroscope’s entire structure when exposed to electrostatic actuation and also to estimate how the structure deflection reacts to the bias voltage. Consequently, a direct current voltage is applied to the ring resonator connected to the semicircular support springs. The deflection of the ring structure for Design I and Design II is in the x-direction as the DC voltage increases, as illustrated in Figure 10. The FEA model shows that Design I has a sudden shift of increase in the displacement value around 40 DC voltages, which shows a pull-in effect. The pull-in effect refers to the electrostatic forces between the electrode and the resonator ring structure. The pull-in effect overcomes the restoring force of the mechanical structure, leading to a sudden increase in displacement as the electrodes snap together. However, once the pull-in occurs, the system enters a nonlinear effect where the relationship between voltage and displacement no longer follows the linear behaviour. When the voltage increases, the electrostatic forces are redistributed and cause a straightforward increase in displacement, resulting in a decrease in overall displacement. Furthermore, the redistribution of electrostatic forces results in an increased electrostatic damping effect. This leads to a higher dissipation of energy as heat or resistance, which ultimately decreases the efficiency of the gyroscope ring structure’s response. Also, there is a case when the structure encounters mechanical stiffness and contact nonlinearities follow a pull-in effect. This also results in alterations to the effective stiffness and constraints that minimized the displacement. Therefore, bias voltages should be lower than the 40 DC voltage. However, Design II has a sharp increment of deflection of about 0.007 µm at 55 DC voltages, which shows that Design II has a slightly higher cut-off bias voltages than Design I. In Design II, the sharp spike observed at 55 V can be explained by the electrostatic pull-in voltage, which is a well-known behaviour in MEMS devices. The electrostatic pull-in voltage occurs when the applied voltage induces an electrostatic force that overcomes the mechanical restoring force of the system. This leads to a sudden increase in displacement or rotation of the MEMS structure. The magnitude of the displacement increases relatively with the increment of the DC voltages for both designs. There is a little discrepancy between the results of the FEA model for both designs, as after 40 DC voltages, there are high values of displacements for Design II when the DC voltages increase. This FEA result assumes that the compliance of the ring mass and semicircular spring beams in that direction is insignificant. As illustrated in Figure 10, these factors marginally minimize the overall stiffness values.

#### 2.2.4. Modal Analysis

The FEA modal analysis is of utmost importance in computational modelling analyses, particularly in determining the vibration characteristics of mechanical structures such as the MEMS vibrating ring gyroscope. The present methodology comprehensively analyses the behavioural patterns and modal frequencies of the MEMS vibrating internal ring gyroscopes. Modal analysis is a powerful technique that find out details about the vibrational behaviour of structures. By discretizing the gyroscope structure into smaller finite elements and solving the resulting equations, this respective method allows for a comprehensive understanding of the vibrational modes and resonance frequencies of the system. This process can obtain a clear and detailed analysis of the vibrating ring gyroscope’s vibrational characteristics. The modal analysis using the Finite Element Method (FEM) was performed by using Ansys 2023 R1 software on both the internal ring gyroscope Designs I and II.

The modal analysis focuses on the two selected designs of vibrating internal ring gyroscopes. This aims to investigate and analyze various aspects of the internal ring gyroscope, including its structural integrity and overall performance. The initial modelling results of the MEMS vibrating internal ring gyroscope provided better mode matching and gyroscope performance parameters than the traditional external ring gyroscope.

Design I consists of a total of eight semicircular beams, each of which is securely connected to the internal ring resonator. The entire vibrating structure is attached and supported by external anchors positioned to the vibrating structure. Design II has the same geometric characteristics. The only difference is the inclusion of more semicircular support springs, which totalled sixteen. The internal ring design strategy effectively employs isolation techniques to mitigate the impact of external vibrations on the vibrating ring structure. The design specifications encompass several key parameters. Firstly, the ring radius is set at 1000 µm, indicating the distance from the centre of the structure to the outer edge of the ring.

**Figure 11:**
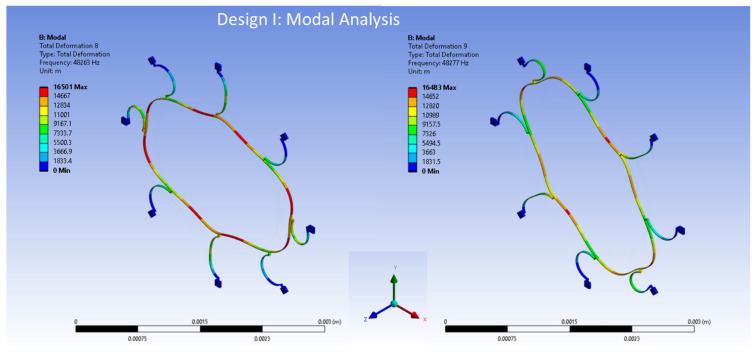
The FEA modal analysis for Design I.

**Figure 12:**
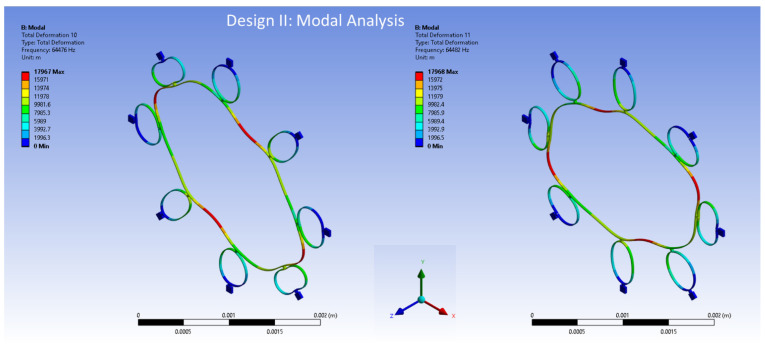
The FEA modal analysis for Design II.

Additionally, the radius of the semicircular support spring is established at 210 µm, signifying the distance from the centre of the structure to the outer edge of the beam. Both the ring and support springs possess a thickness of 10 µm. Lastly, the structure’s height is 30 µm, describing the vertical distance from the base to the top of the structure. The modal analysis results of the elliptical mode of vibrations are shown in Figure 11 for Design I and in Figure 12 for Design II.

The frequencies obtained from the model for the internal ring Design I, as depicted in Figure 11, are 48,263 Hz and 48,277 Hz for mode 1 and mode 2, respectively. The mode mismatch is recorded at 14 Hz. The internal ring design shows better mode-matching results, which is crucial for high-performance devices.

The resonance frequencies resulting from the model for the internal ring Design II, as shown in Figure 12, are 64,476 Hz and 64,482 Hz for mode 1 and mode 2, respectively. The mode mismatch for Design II is recorded only at 6 Hz. This shows that including more semicircular support springs increases the resonance frequency and minimizes the mode mismatch between two identical elliptical modes of vibrations.

#### 2.2.5. Harmonic Analysis

**Figure 13:**
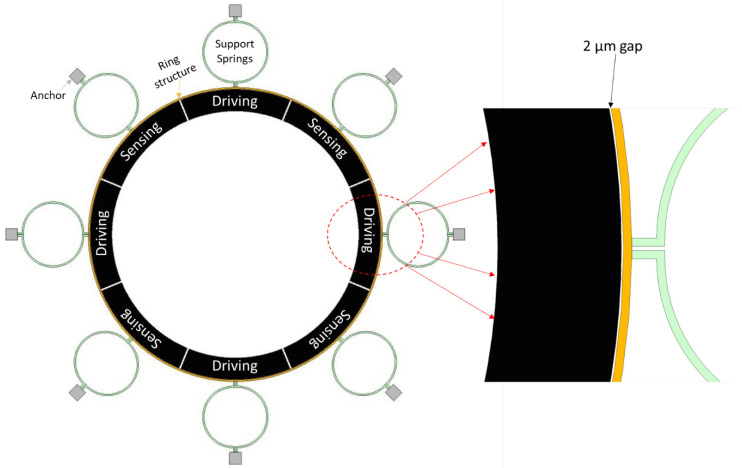
A schematic representation of the electrode scheme for electrostatic actuation with red dotted arrows show enlarge view of the driving electrode.

A comprehensive analysis of the dynamic characteristics of the proposed MEMS vibrating internal ring gyroscopes should include a harmonic response analysis. This analysis investigates the frequency-dependent behaviour of the gyroscope and ultimately determines its mechanical sensitivity. The internal ring resonator is subjected to a harmonic force in the directions of the X and Y axes through the actuation voltage consisting of a DC voltage of 20 V and an alternating current (AC) component of 0.5 V. This actuation is achieved using driving electrodes placed inside the ring resonator. A schematic representation of the electrode configuration with the internal ring gyroscope is shown in Figure 13.

The electrical design setup was demonstrated in [39], where the actuation equation was derived for the vibrating ring gyroscope.

(29) $$F_{b-g}=2\frac{\varepsilon_{o}\;w\;h\;N}{{y_{o}}^{2}}V_{DC}v_{AC}$$

It is observed that the AC voltage values are considerably smaller in comparison to the DC voltage. Consequently, the nonlinear effects arising from electromechanical coupling can be disregarded, allowing for the execution of a prestressed harmonic analysis to examine the harmonic behaviour of the gyroscope. An initial static analysis is conducted using the applied direct current (DC) voltage following the experimental procedure. This is followed by a comprehensive harmonic analysis, considering the pre-existing stress conditions and utilizing the applied alternating current (AC) excitation.

**Figure 14:**
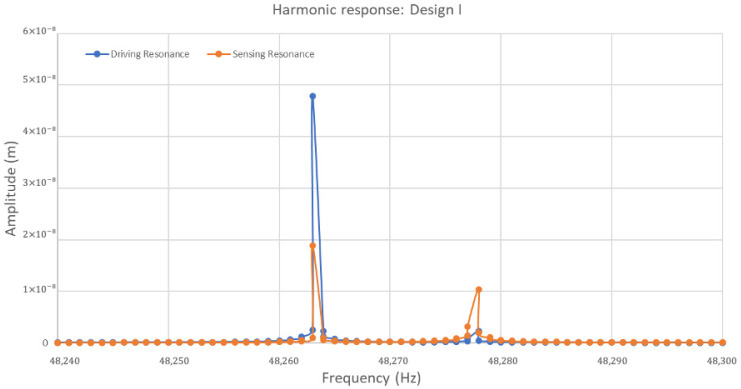
A harmonic response analysis for the MEMS vibrating internal ring gyroscope Design I.

The frequency responses of the ring structure in the drive and sense direction for Design I are presented in terms of amplitude in Figure 14. A modal analysis for Design I showed two resonance frequencies of elliptical modes at 48,263 Hz and 48,277 Hz, respectively. In the context of harmonic response, the first peak value of driving resonance frequency was 48,263 Hz with an amplitude of 0.05 µm, and the second one was 48,277 Hz with a very small amplitude. On the other hand, in sensing resonance frequency, the highest peak value observed at 48,263 Hz is primarily selected for the driving resonance frequency, and the second peak is observed at 48,277 Hz with an amplitude of 0.01 µm. The sensing resonance shows a lower peak amplitude, which shows the gyroscope system’s response to sensing is less pronounced than driving vibrations at the resonant frequency. The quality factors for driving and sensing modes were recorded at 3400 for each.

**Figure 15:**
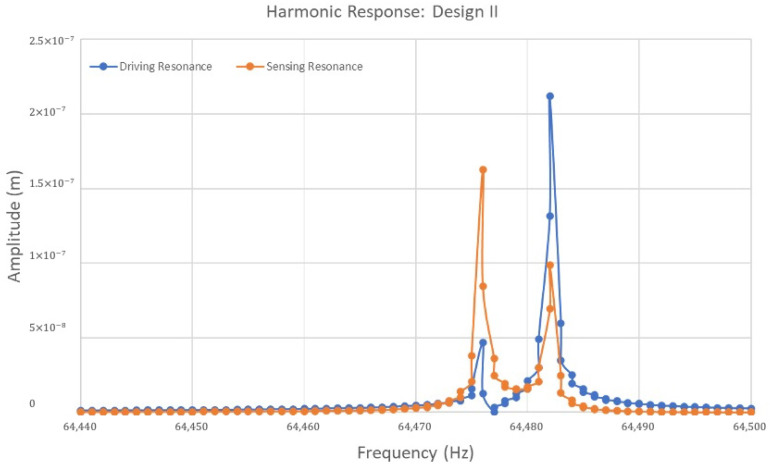
A harmonic response analysis for the MEMS vibrating internal ring gyroscope Design II.

For Design II, with its sixteen semicircular support springs, the harmonic response of the ring structure is presented as the amplitude in Figure 15. These responses are presented in the drive and sense direction. A modal analysis was conducted for Design II in the presence of two identical elliptical modes of resonance frequencies. These frequencies were observed at higher values of 64,476 Hz and 64,482 Hz, respectively. In harmonic response analysis, it is observed that the highest peak value of the driving resonance frequency occurs at 64,482 Hz, exhibiting a higher amplitude of 0.21 µm. Subsequently, a lower peak in driving resonance frequency is observed at 64,476 Hz with an amplitude of less than 0.04 µm. In sensing resonance frequency, it is important to notice that the highest peak value of an amplitude of 0.02 µm was recorded at a frequency of 64,476 Hz. This frequency is predominantly chosen as the sensing resonance frequency. The second peak of sensing resonance was observed at a slightly higher frequency of 64,482 Hz with an amplitude of 0.01 µm. The peak values in Design II are sharper and more pronounced, especially in driving resonance frequency, which reaches a higher amplitude than Design I.

Including more semicircular support springs contributes to a more refined separation between driving and sensing resonance frequencies. This is quite important for the vibrating ring gyroscope’s ability to detect rotation without interference from other sources of vibration. The sharp peaks in both designs indicate that the gyroscope system possesses high Q factors. The quality factor Q_D_ for the driving mode was recorded to be 10,747 and the quality factor Q_S_ for the sensing mode was recorded to be 10,746. The amplitude outside the resonant peaks is minimal and can easily be filtered out for higher performance.

### 2.3. Harsh Conditions

The advancement of MEMS technology has significantly revolutionized the field of inertial sensors. MEMS vibrating ring gyroscopes have emerged as an integral part of inertial measurement units (IMU) for various applications. These gyroscopes are highly suitable for advanced integration into various smart devices. The increasing demand for these inertial sensors in various harsh conditions significantly challenges their reliability and performance. The reliability of MEMS gyroscopes heavily depends on their ability to maintain the highest levels of accuracy and durability, particularly when exposed to harsh conditions such as extreme temperatures. These conditions can cause many reliability issues, including problems with material degradation and electronic malfunction.

This section emphasizes mode matching and temperature analysis to analyze the MEMS vibrating ring gyroscope’s performance in harsh conditions. Mode matching is a fundamental technique to synchronize the driving and sensing resonant frequencies to achieve the optimal performance of the gyroscope. The impact of temperature fluctuation is quite important. The temperature fluctuations develop thermal stresses and strains in the gyroscope which further affect the performance. The degradation of the material and mode mismatching effects due to temperature variations are investigated in this section.

#### 2.3.1. Temperature Analysis

The most critical challenge in designing MEMS vibrating ring gyroscopes is the variability in the gyroscope’s performance parameters due to environmental changes and fabrication imperfections. These issues can lead to a mismatch in the two operating resonance frequencies. This results in a discrepancy between the frequencies of the driving and sensing modes. The mode mismatching can be minimized by prefabrication design geometry modifications and electrostatically by tuning electrodes. This section presents a design modelling analysis to counteract fluctuations in device operating temperature. It specifically highlights the enhancements in performance achieved through two closely operating resonance frequencies. Ensuring performance stability is important in the field of MEMS gyroscopes. As discussed earlier, even minor discrepancies between the driving and sensing modes’ resonance frequencies lead to significant inaccuracies in the output signal gain. Temperature fluctuations can potentially induce changes in the resonant frequency of the drive and sense modes in MEMS vibrating ring gyroscopes, thereby impacting their overall performance. Therefore, it is essential to consider the impact of thermal fluctuations on the performance evaluation of the MEMS vibrating ring gyroscope. Temperature fluctuations can lead to alterations in Young’s modulus, thermal expansion or contraction, and thermally induced stresses. These changes can affect the stiffness matrix, resulting in variations in the resonant frequency values and a decline in the performance of the device. The modelling and simulation of the thermal-induced effects on the behaviour of the proposed MEMS vibrating ring gyroscopes are presented below.

**Material Degradation:** The proposed MEMS vibrating ring gyroscopes were designed to utilize the simple silicon-on-insulator (SOI) microfabrication process, which involves using silicon as the primary material for the structure. Temperature fluctuation has an impact on the properties of silicon, especially its elastic properties. Variations in the resonance frequency of the MEMS gyroscope can occur due to the temperature fluctuations that bring changes in the Young’s modulus of silicon material. An equation that describes the influence of temperature on Young’s modulus of silicon is given below as Equation (30) [42].

(30) $$E=E_{0}-BT\exp\left(\frac{-T_{0}}{T}\right)$$

**Figure 16:**
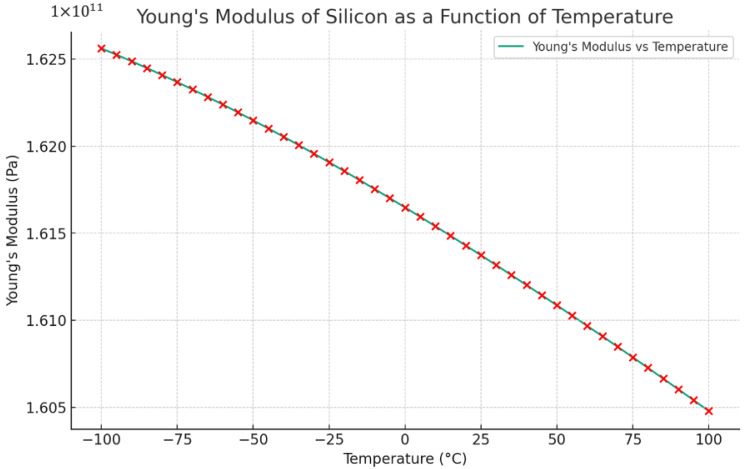
Temperature fluctuations affect the Young’s modulus of silicon material and red cross shows values at different temperatures.

Here, $E_{0}$ represents the Young’s modulus at absolute zero temperature. T refers to the temperature. B and $T_{0}$ are constants that rely on the Grueneisen parameter, Debye temperature, Anderson–Grueneisen parameter, and material volume at 0 K. The calculated values for the B and $T_{0}$ of silicon material are 15.8 MPa and 317 K, respectively [42]. The Young’s modulus value of silicon for the temperature range from −100 °C to 100 °C, relevant to the proposed MEMS vibrating ring gyroscope, is shown in Figure 16. The results indicate that the temperature variation has a negligible impact on Young’s modulus and stiffness variation for the MEMS vibrating ring gyroscope within the specified operating temperature range. A temperature range from −100 °C to +100 °C was chosen based on the operational environments expected for the MEMS vibrating ring gyroscopes, such as space and high-temperature industrial applications. This range encompasses the extreme temperatures that the gyroscopes might encounter during operation, ensuring that the designs are robust enough to withstand significant thermal fluctuations.

**Thermal Impact on MEMS Vibrating Internal Ring Gyroscopes:** Temperature variations within the gyroscope device can cause expansion and contraction in the microstructure in addition to changes in the material properties. Thermal deformation in the MEMS vibrating ring gyroscopes can cause changes in the gap spacing between the ring structure and capacitive electrode. Furthermore, temperature variations may lead to thermal stresses and change the dimensions of the support springs and the whole gyroscope structure. An FEA model is used to conduct a thermal analysis to accurately measure the changes in resonant frequency caused by thermal deformation and thermally induced stresses for the proposed Design I and Design II vibrating internal ring gyroscopes. The gyroscopes are intended to operate within a temperature range from −100 °C to 100 °C. Initially, a uniform temperature T is assigned to all structural elements, ranging from −100 °C to 100 °C. A static analysis is performed with prestress effects enabled, considering the ambient temperature T = 22 °C as the reference temperature.

**Figure 17:**
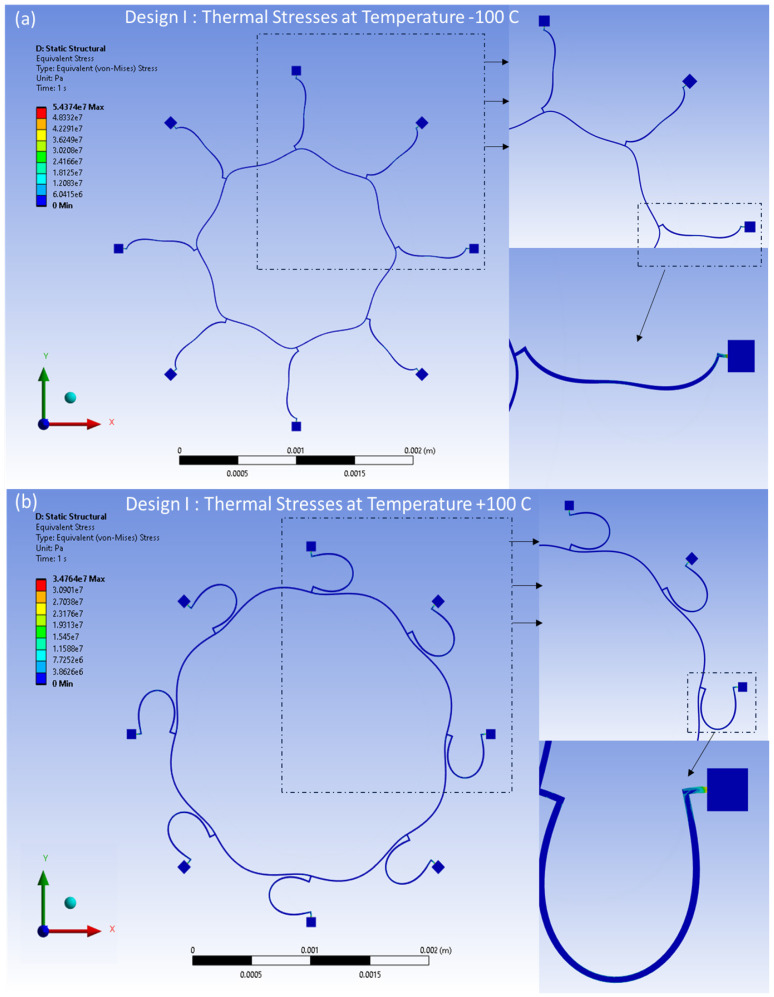
Design I: thermal stresses developed at (**a**) −100 °C, (**b**) +100 °C.

**Figure 18:**
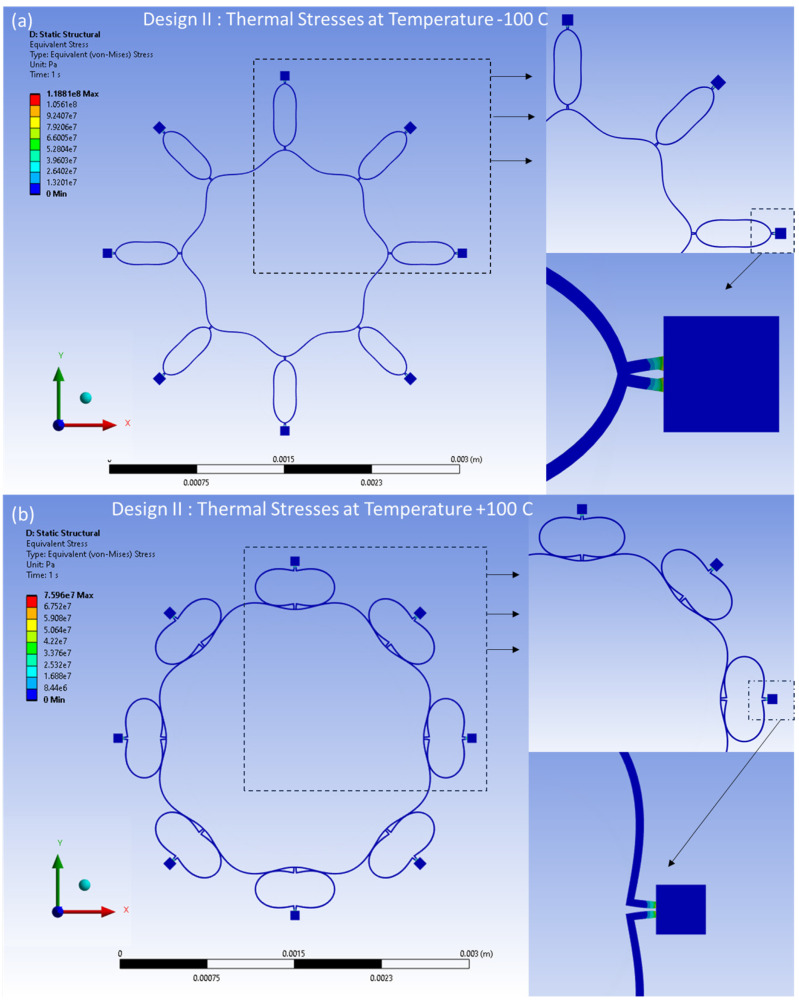
Design II: thermal stresses developed at (**a**) −100 °C, (**b**) +100 °C.

The distributed thermal stresses resulting from the FEA model-based static analysis are shown in Figure 17a,b for Design I and in Figure 18a,b for Design II. These stresses are induced by thermal effects and are observed at temperatures between −100 °C and 100 °C, respectively. A notable accumulation of thermal stresses is observed in the vicinity of the semicircular support springs attached to the externally placed anchors. Conversely, minimal thermal stresses are observed in the ring and the remaining semicircular support springs portions of the structure.

**Figure 19:**
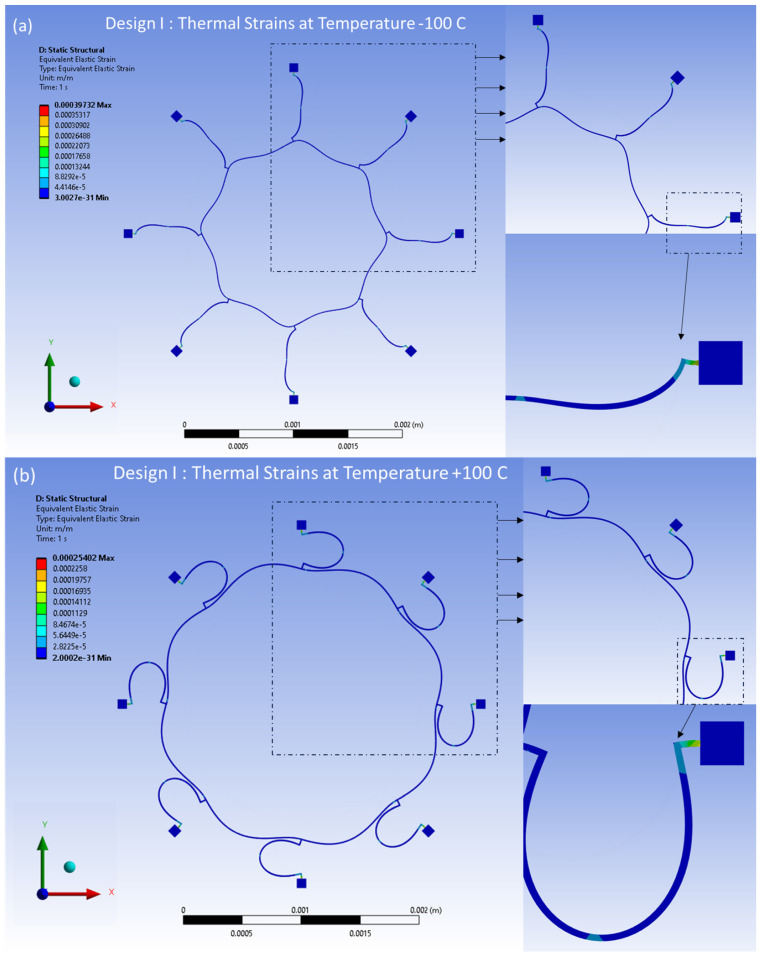
Design I: thermal strains developed at (**a**) −100 °C, (**b**) +100 °C.

**Figure 20:**
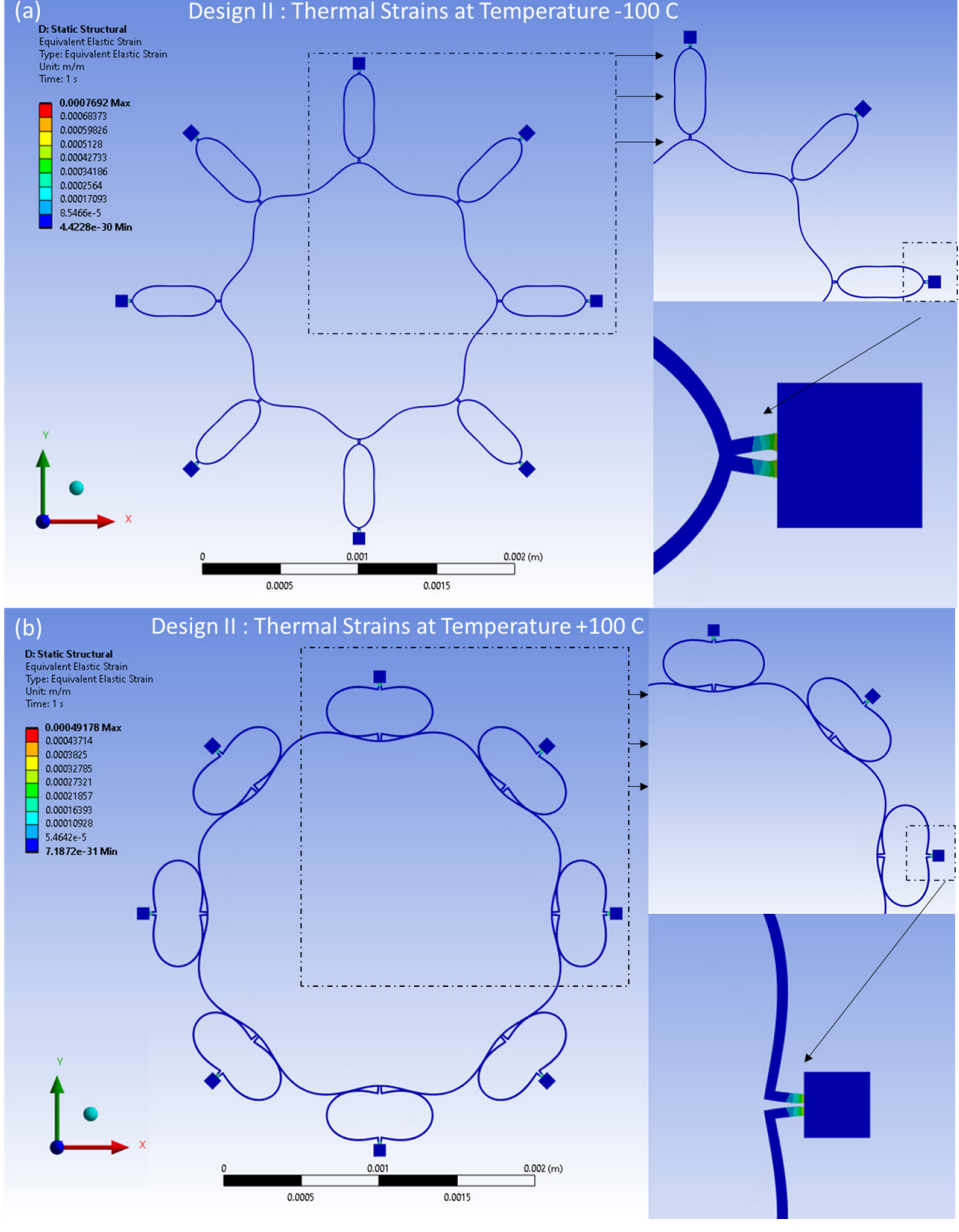
Design II: thermal strains developed at (**a**) −100 °C, (**b**) +100 °C.

Similarly, the distributed thermal strains resulting from the FEA model are shown in Figure 19a,b for Design I and in Figure 20a,b for Design II. These strains are induced by thermal effects and are observed at temperatures between −100 °C and 100 °C, respectively. The distributed strains are observed mainly around the semicircular support springs attached to the externally placed anchors. However, minimal thermal strains are observed in the ring and the remaining semicircular support spring area of the structure.

**Figure 21:**
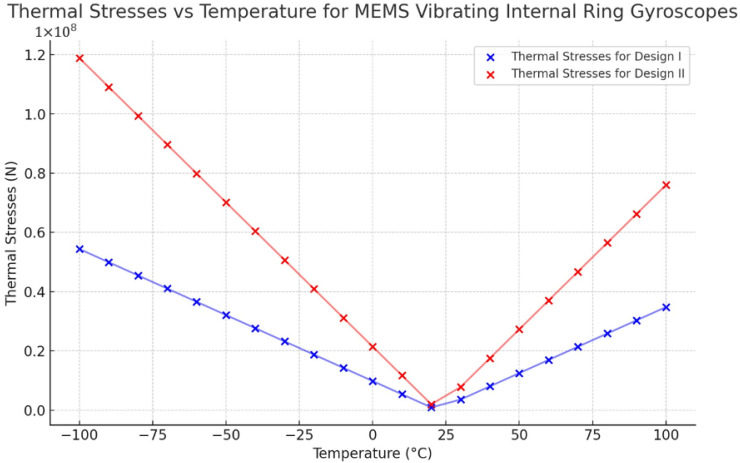
Thermal stresses vs. temperature changes for Design I and Design II.

The thermal analysis modelling results clearly show that Design II is more robust than Design I. The thermal results indicate that the resonant frequency shift in both the drive and sense modes is expected to exhibit variations in response to fluctuations in environmental temperature. However, the symmetric design structure of the vibrating ring gyroscope can cause uniform shifts in both the resonant frequencies. The findings of the maximum value of thermal stresses resulting from temperature fluctuations ranging from −100 °C to 100 °C are presented in Figure 21 for both Designs I and II. The observed values increase when the magnitude of the temperature differential is greater than the ambient room temperature.

**Figure 22:**
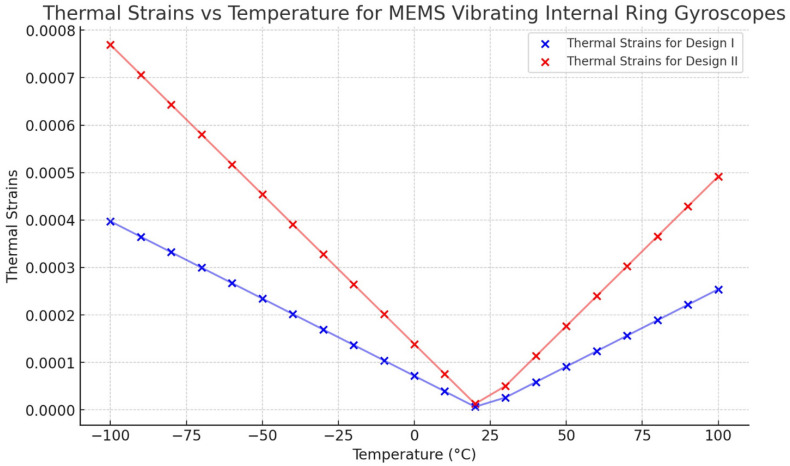
Thermal strains vs. temperature changes for Design I and Design II.

The thermal strain result is presented in Figure 22. The results indicate a linear relationship between thermal strain and temperature changes for both designs. However, Design II exhibits a higher strain value than Design I. The strain results also affect the shift of the resonant frequencies uniformly.

**Figure 23:**
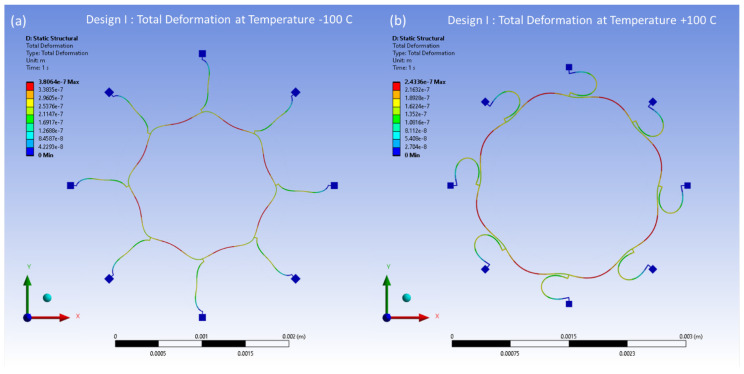
Design I: thermal deformation vs. temperature changes. (**a**) Deformation at −100 °C. (**b**) Deformation at +100 °C.

**Figure 24:**
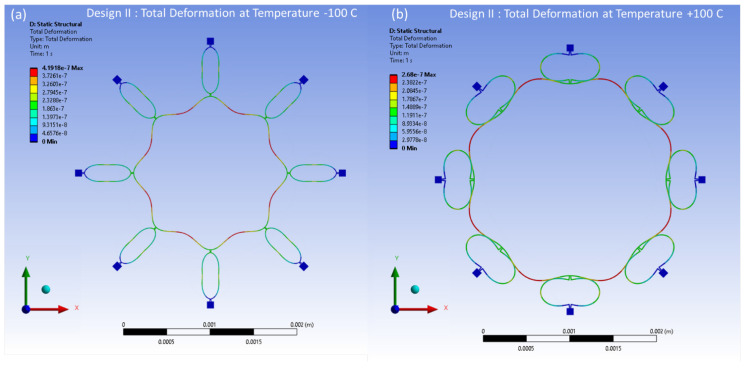
Design II: thermal deformation vs. temperature changes. (**a**) Deformation at −100 °C. (**b**) Deformation at +100 °C.

The thermal deformations of the reference temperature of 22 °C have been modelled within the temperature range from −100 °C to 100 °C. The thermal deformation results at between −100 °C and 100 °C are illustrated in Figure 23 and Figure 24 for Design I and Design II, respectively. In Figure 23, the structural components of the ring and semicircular springs experience contraction due to thermal deformation occurring at a temperature of −100 °C in Design I. This contraction reaches a peak value of 0.38 μm. On the other hand, when the temperature reaches 100 °C, the vibrating structures, including the ring and semicircular support springs, undergo expansion. The ring and spring structure exhibit a maximum deformation of 0.24 μm. In Figure 24, the ring and semicircular springs structural components experience contraction up to 0.41 μm at a temperature of −100 °C in Design II. When the temperature reaches a peak of 100 °C, the ring and semicircular support spring structure undergoes an expansion of up to 0.27 μm.

**Figure 25:**
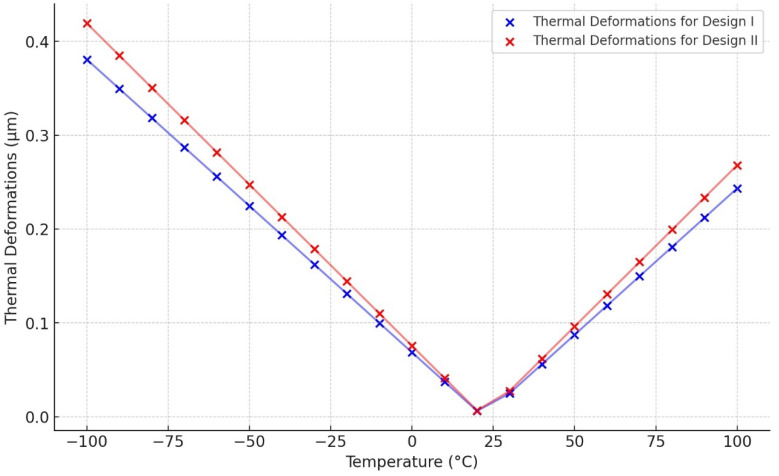
Thermal deformation vs. temperature changes for MEMS vibrating internal ring gyroscopes.

The thermal deformation results show that both designs have a uniformly distributed deformation across the ring and semicircular support spring structure. The temperature range is quite wide from −100 °C to 100 °C. Furthermore, Figure 25 confirms a linear relationship between the deformation and the temperature changes for the MEMS vibrating ring gyroscope due to its symmetric design nature. The thermal deformation value increases when the temperature difference is greater than the room temperature.

#### 2.3.2. Mode Matching

Mode matching is an essential parameter in MEMS vibrating ring gyroscopes for optimal and higher performances. This includes matching the resonance frequencies of the driving and sensing modes. To achieve mode matching prior to fabrication, include the precision required in microfabrication procedures plus predesign modification modelling. Variations in the gyroscope structural dimensions are frequently caused by microfabrication errors such as sidewall angle and critical dimension losses. These errors cause a frequency split between these modes. Overcoming this error requires improved prefabrication procedures, design feature variation research, and the use of automatic control systems. Tuning electrodes are also essential for adjusting the mode mismatch of the resonance frequencies. The temperature fluctuations also contribute to the mode mismatch.

**Figure 26:**
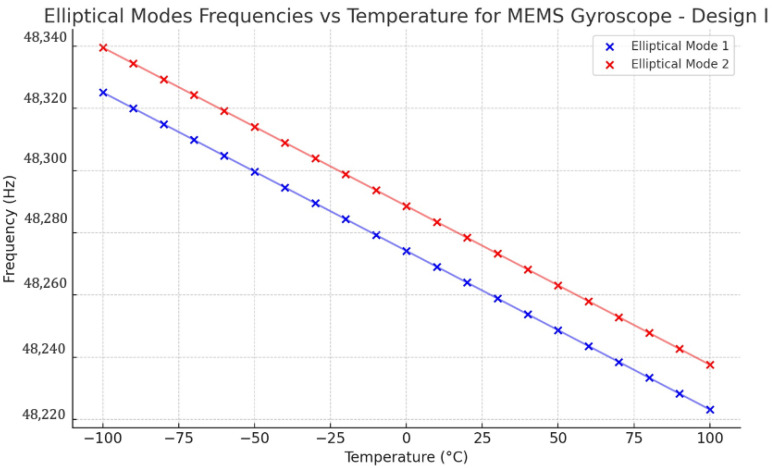
Design I: effect of temperature changes on resonance frequencies.

It has been observed that temperature fluctuations significantly affect the performance of MEMS gyroscopes. The thermal deformations and stresses developed from these temperature-induced variations occur within the ring and support spring structures. As a result, modifications are introduced to the drive and sense modes’ frequencies. Based on the elevated thermal deformations and stresses detected in the ring and support springs, it is rational to expect temperature operating variations to have a more pronounced effect on the resonant frequency. To find out the mode mismatch between the two identical elliptical modes of vibrations in both designs, a parametric study has been conducted to observe the thermal effect on the mode matching. The resonance frequency drop can be observed in Figure 26 for Design I, showing a linear drop from −100 °C to 100 °C. The mismatch between the resonant frequencies is roughly 15 Hz. At room temperature 22 °C, the resonance frequencies of elliptical mode 1 and mode 2 were observed at 48,263 Hz and 48,277 Hz, respectively.

**Figure 27:**
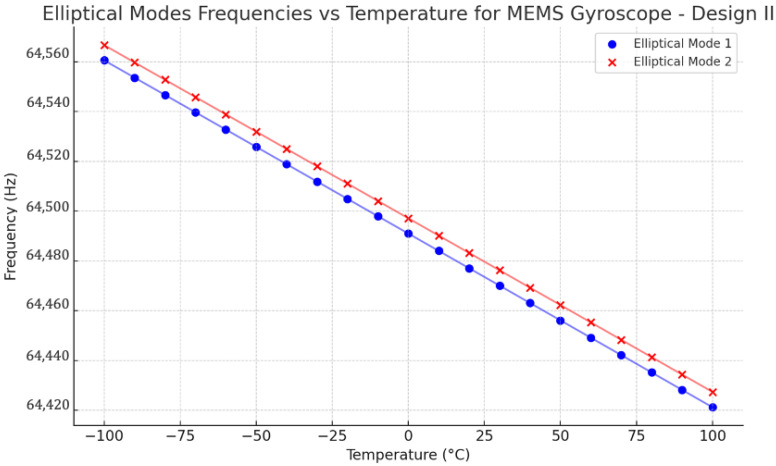
Design II: effect of temperature changes on resonance frequencies.

Design II was modelled with a pre-thermal analysis to analyze the two identical elliptical modal frequencies against the temperature range from −100 °C to 100 °C. The obtained result is shown in Figure 27. The respective modal frequencies consistently change with the temperature changes. At 22 °C, the elliptical modal frequencies, mode 1 and mode 2, were observed at 64,476 Hz and 64,482 Hz, respectively. The mode mismatch between modal frequencies was observed at 6 Hz, which shows a better result than Design I.

**Figure 28:**
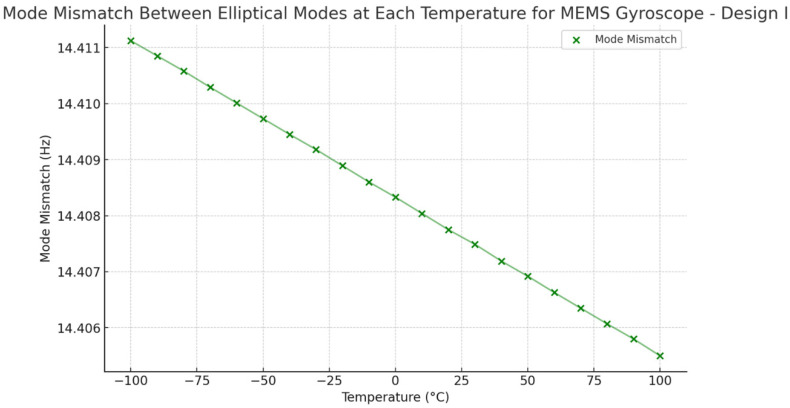
Design I: mode mismatch resonance frequencies.

**Figure 29:**
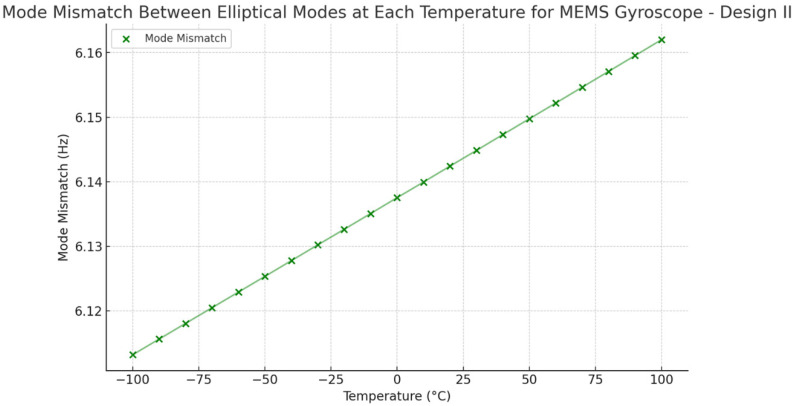
Design II: mode mismatch resonance frequencies.

To compare the mode mismatch for both the MEMS vibrating internal ring gyroscope designs, Design I shows a higher mode mismatch value. The mode mismatch value is around 14 Hz, as shown in Figure 28. On the other hand, Design II shows a low mode mismatch value of roughly 6 Hz, as seen in Figure 29. The mode mismatch value is almost consistent for Design II. It clearly shows that Design II has a lower mode mismatch value and higher resonance frequencies than Design I.

The modelling results of the temperature analysis and effect of mode mismatching were analyzed in two designs of MEMS vibrating internal ring gyroscopes, Design I and Design II. Both designs have the same design features; the only difference is that Design II has more semicircular support springs than Design I. In Design II, the semicircular springs were placed as an opposite pair, and a total of sixteen support springs were used. Adding more springs increases the desired elliptical modes’ resonance frequencies to more than 60 kHz. The increased resonance frequency and placement of more springs outside of the ring resonator made the gyroscope more stable when exposed to harsh conditions. The thermal stress distribution in the ring resonator and semicircular springs is higher in Design II than in Design I. This suggests that Design II has more vibrating structures than Design I, which increases the thermal stresses and strains in Design II. However, the thermal deformations are approximately the same in both designs. The results show that Design II performs better than Design I when experiencing harsh conditions. The mode mismatch results obtained in Design II are significantly less. The mode mismatch value remains the same around 6 Hz within the temperature range from −100 °C to 100 °C. Design I shows a mode mismatch value of around 14 Hz, which is more than double that of Design II. However, both vibrating internal ring gyroscope designs show a lower mode mismatch value than the traditional external ring gyroscope, which resulted in 31 Hz, as modelled in [39]. The comparison results for mode matching two internal vibrating ring gyroscopes and an external ring gyroscope are shown in Table 3.

## 3. Conclusions

We have successfully investigated the design and modelling of two MEMS vibrating internal ring gyroscopes in consideration of the harsh environmental conditions. The paper demonstrated the performance enhancement of the gyroscopes’ sensitivity, precision, and thermal stability through innovative design modifications.

The comprehensive analysis of the two proposed designs, Design I with eight semicircular support springs and Design II with sixteen semicircular support spinrgs, presented the key details as listed below.

Enhanced Mode Matching: Design II achieved significantly better mode matching with a mode mismatch of only 6 Hz as compared to 14 Hz in Design I.

Thermal Stability: The thermal analysis demonstrated that Design II offers better performance under extreme temperature conditions. Design II shows more uniform thermal stress and strain distribution throughout the structure. It is essential for maintaining consistent performance in harsh environments.

Higher Resonance Frequencies: Design II exhibited higher resonance frequencies, approximately 64 kHz, than Design I, approximately 48 kHz. This indicates improved dynamic performance and robustness against external vibrations and temperature fluctuations.

Electrostatic and Harmonic Analysis: Both designs were subjected to electrostatic and harmonic analyses to evaluate their behaviour under varying DC voltages and harsh temperature conditions. Design II showed higher cut-off bias voltages and more pronounced peak amplitudes in the harmonic response analysis.

These research findings suggest that the MEMS vibrating internal ring gyroscope with additional support springs, specifically Design II is more suitable for harsh conditions such as high-temperature applications. Improved mode matching, thermal stability, and higher resonance frequencies make Design II a better option for advanced inertial measurement systems. Future research will focus on further design optimization and fabrication processes to characterize the proposed Design II for challenging conditions.

## Figures and Tables

**Table 1:** Design parameters of Design I: MEMS vibrating internal ring gyroscope.

	Design Parameters	Value (µm)
1	Internal ring radius	1000
2	Structure height	100
3	Ring thickness	10
4	Number of Semicircular springs	8
5	Semicircular spring radius	210
6	Semicircular spring thickness	10
7	Anchor area	80 × 80

**Table 2:** Design parameters of Design II: MEMS vibrating internal ring gyroscope.

	Design Parameters	Value (µm)
1	Internal ring radius	1000
2	Structure height	100
3	Ring thickness	10
4	Number of semicircular springs	16
5	Semicircular spring radius	210
6	Semicircular spring thickness	10
7	Anchor area	80 × 80

**Table 3:** A comparison of results between vibrating internal and external ring gyroscopes.

Frequency (Hz)	Internal Ring Design II	Internal Ring Design I	External Ring Design	Ring Radius (µm)	Ring Thickness (µm)	Spring Radius
Mode 1	64,476 Hz	48,263 Hz	40,241 Hz	1000	10	200
Mode 2	64,482 Hz	48,277 Hz	40,272 Hz	1000	10	200
Mode Mismatch	6 Hz	14 Hz	31 Hz	1000	10	200
Total Springs	16	8	8	-	-	-

## Data Availability

The data presented in this article are available on request from the corresponding author.
